# The role of sialidase Neu1 in respiratory diseases

**DOI:** 10.1186/s12931-024-02763-9

**Published:** 2024-03-19

**Authors:** Shiran Mei, Dingding Li, Aoyi Wang, Guoxue Zhu, Bingwen Zhou, Nian Li, Yi Qin, Yanliang Zhang, Shujun Jiang

**Affiliations:** 1 Nanjing Hospital of Chinese Medicine Affiliated to Nanjing University of Chinese Medicine, Nanjing, China; 2 Nanjing Research Center for Infectious Diseases of Integrated Traditional Chinese and Western Medicine, Nanjing, Jiangsu China

## Abstract

Neu1 is a sialidase enzyme that plays a crucial role in the regulation of glycosylation in a variety of cellular processes, including cellular signaling and inflammation. In recent years, numerous evidence has suggested that human NEU1 is also involved in the pathogenesis of various respiratory diseases, including lung infection, chronic obstructive pulmonary disease (COPD), asthma, and pulmonary fibrosis. This review paper aims to provide an overview of the current research on human NEU1 and respiratory diseases.

## Introduction

Respiratory disease refers to a group of disorders that affect the respiratory system, including the lungs, airways, and other structures involved in breathing. These diseases can range from mild conditions such as the common cold to more severe illnesses like pneumonia and chronic obstructive pulmonary disease (COPD). Previously, much attention has been paid to the role of Neu1from viral and bacterial in human respiratory diseases. In recent years, there has been growing interest in the role of human Neuraminidases 1 (Neu1), a sialidase enzyme, in the development and progression of respiratory diseases [1–10].

Neu1, as the most abundant sialidase located in lysosomes and on the cell membrane of mammal, is an enzyme that plays a crucial role in the removal of sialic acid residues from glycoproteins and glycolipids. Sialic acids are important components of cell surface molecules and are involved in various cellular processes, including cell adhesion, signaling, and immune response. The production, secretion, and activity of sialidase can also be regulated by proteins that bind to sialidases, especially protective protein/cathepsin A (PPCA) binding to NEU1. The binding of glycosidase β-Gal, PPCA and NEU1 formed lysosomal multiprotein complex, which regulate the intralysosomal catabolism of sialylated macromolecules [11]. Neu1, PPCA and elastic binding protein (EBP) are together assembled the elastin receptor complex (ERC), which modulated the assembly of elastic fibers [12]. Dysregulation of Neu1 activity has been implicated in several diseases, including cancer, metabolism disorder, neurodegenerative disorders, and respiratory diseases. It has been proved that NEU1 is expressed in human airway smooth muscle cells, epithelial and microvascular endothelial cells, fibroblasts, and in the lungs of patients with idiopathic pulmonary fibrosis (IPF) [1–6]. Neu1 was upregulated in lung tissues of patients with COPD and asthma, suggesting that it may play a role in the development of these diseases [1, 2]. It has been reported that Neu1 was upregulated in airway smooth muscle cells from patients with asthma, and inhibition of Neu1 reduced airway hyperresponsiveness in mice with asthma [3, 4]. Consistent with this, Neu1 expression was increased in lung tissue samples from patients with interstitial lung disease (ILD), correlated with increased inflammation and mucus production in the airways [5, 6]. Above results indicated that the abnormally high expression of Neu1 was correlated with the high immune response in human respiratory diseases.

Neu1 has also been implicated in the pathogenesis of pulmonary fibrosis, a progressive and often fatal lung disease. Previous study demonstrated that Neu1 was upregulated in lung tissues of patients with pulmonary fibrosis, and that inhibition of Neu1 reduced lung fibrosis in a mouse model of the disease [5–7]. While the exact mechanisms by which Neu1 contributes to lung disease are not yet fully understood, it is believed that Neu1 plays a role in the regulation of cell signaling pathways, inflammation, and extracellular matrix remodeling. Thus, targeting Neu1 may represent a promising therapeutic approach for the treatment of diverse respiratory diseases.

**Fig. 1:**
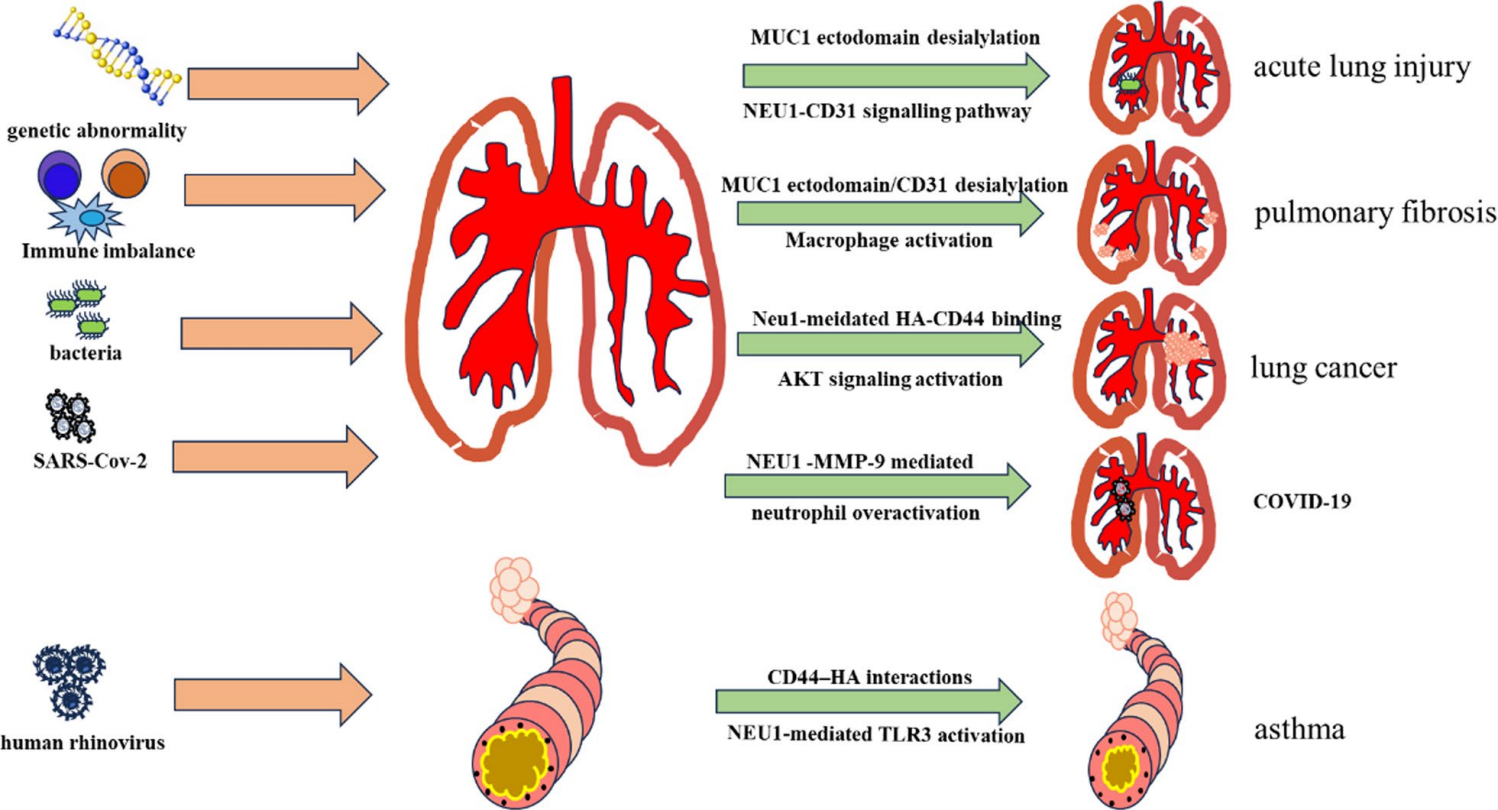
Schematic view of the role of the Neu1 in respiratory disease

In this review, we used keywords such as “Neu1”, “sialidase 1”, “respiratory disease”, “lung infection”, “COPD”, “asthma”, and “pulmonary fibrosis” to identify relevant articles on PubMed. Here, we will summary the recent understanding of Neu1 and its relevance for pulmonary health and disease (Fig. 1), illustrating its potential clinical application.

## NEU1 and lung injury

Airway epithelial cells (EC) express sialylated receptors that recognize bacterial pathogens, and mediate its interaction with host cells. P. aeruginosa (Pa) as a Gram-negative, flagellated, opportunistic human pathogen, adhered to ECs by its flagellin interacted with cell surface sialoprotein transmembrane mucin 1 (MUC1). Simeon and colleague found that flagellin from Pa could promote bacterial adhesion to and invasion of ECs by Neuraminidase 1 (NEU1)-dependent MUC1 ectodomain desialylation in vitro [8]. Subsequently, they also verified that Neu1 provoked the shedding of MUC1-ED from airway, which suppressed Pa lung infection in BALB/c mice [9]. Recently, Simeon’ team reported that the concentration of desialylated MUC1-ED and flagellin expression in Pa were dramatically increased in bronchoalveolar lavage fluid (BALF) harvested from Pa-infected patients [10], this indicated that measurement of MUC1-ED in BALF levels might serve as a guide for antibiotic therapy in patients with Pa-infections. In summary, different in vivo experiments confirmed that NEU1 might modulate the desialylation of human ECs receptors to resist the pulmonary bacterial infections.

NEU1 highly expressed in vascular endothelia of different human tissues. It has been reported that NEU1 overexpressed in human lung microvascular EC (HMVEC-Ls) inhibited their migration into wound [1]. Subsequent research found that NEU1 mediated inhibition of angiogenesis in human pulmonary microvascular ECs (HPMECs) through desialylation of CD31 [13]. The results of further research manifested that NEU1-CD31 interaction might drive by src family kinases during angiogenesis process for postconfluent HPMECs. According to a in vivo study, Neuraminidase promoted desialylation in polymorphonuclear leukocytes, potentiated inflammatory response and vascular collapse in LPS-induced acute lung injury [14]. These studies indicated that NEU1 has negative effect on the pulmonary reprogram after acute injury.

## NEU1 and pulmonary fibrosis

As typical feature of pulmonary fibrosis, epithelial abnormalities, vascular remodeling, abnormal wound healing and angiogenesis caused histopathological changes and functional decline of the lungs. Numerous evidence implicated various genes, including cell surface receptors, their ligands, extracellular matrix molecules and downstream signaling pathways, were involved in pathogenesis of pulmonary fibrosis [15, 16]. The imbalance between pro-inflammatory and regulatory immune cell subsets is a cardinal cause of pulmonary fibrosis. The preponderance of evidence demonstrated that elevated NEU1 expression regulated the desialylation and activity of receptors (such as platelet-derived growth factor receptor and Toll like receptors) in epithelial and endothelial cells involved in the development of pulmonary fibrosis [17, 18]. It has been verified that elevated expression of NEU1 sialidase provokes pulmonary fibrosis by aggravated lymphocytic infiltration and collagen accumulation in human [3]. Neu1-dependent CD31 desialylation modulated the capillary-like tube formation, which was correlated with pulmonary angiogenesis in lungs of patient with IPF [12, 19].

Selective inhibition of Neu1 attenuated bleomycin-induced lung inflammation and fibrosis by mediated desialylation of Muc1 ectodomain [8]. Recent study showed that sialidases was higher in male compared to female mice induced by bleomycin, high NEU1 expression closely correlated with CD11b^+^ macrophages in BALF from lung tissue of bleomycin induced female mice. Both NEU2 and NEU3 levels were associated with some inflammation and fibrosis markers independent on gender [20]. Meanwhile another study found that abnormally serum sialidase NEU3 level promoted fibrosis through serum amyloid P (SAP) desialylation acceleration and IL-10 accumulation by PBMC in idiopathic pulmonary fibrosis (IPF) patients [21]. Consistent with this, pulmonary fibrosis was strongly attenuated in bleomycin-induced Neu3 knockout mice [22]. Recent research confirmed that inhibition of NEU3-mediated TGF-β1 activation might rescued the lung injury caused by bleomycin [9]. These studies suggested that both Neu1 and Neu3 might have pronounced effects on pulmonary fibrosis process.

## NEU1 and asthma

Asthma is an airway disease, characterized with increased serum levels of IgE and inflammation being caused by elevated levels of T(H)2-type cytokines. T helper 2 (Th2) cells are abundant in type 2 (T2) asthma. CD4^+^ T cells accumulated in the airway are associated the development of asthma [23]. The cell surface adhesion receptor cluster of differentiation 44 (CD44) as a highly glycosylated molecule, interacted with hyaluronic acid (HA), to participate in the airway accumulation of CD4^+^ T cells in murine model of asthma [3, 4]. Th2-mediated airway inflammation was caused by CD44–HA interactions through Neu1-meidated desialylation in the airway of asthma mouse model. Another study has demonstrated Neu1 sialidase activity in respiratory airway epithelia regulated EGFR- and MUC1-dependent signaling and bacterial adhesion in vitro [2]*.* It has been reported that NEU1 interacted with Toll like receptors (TLR2, 3, 4) and activated TLR signaling [24]. TLR3 could recognize human rhinovirus (HRV) RNA and highly expressed in lung epithelial cells post-HRV infection [25]. Martin and colleague demonstrated that higher cytokine expression in asthmatics is correlated with decrease of NEU1-mediated TLR3 activation in asthmatic airway cells after HRV infection in children [26]. These reports supported that Neu1 might have the potential for further applications in asthma prevention or treatment.

## NEU1 and tumors of lung

**Fig. 2:**
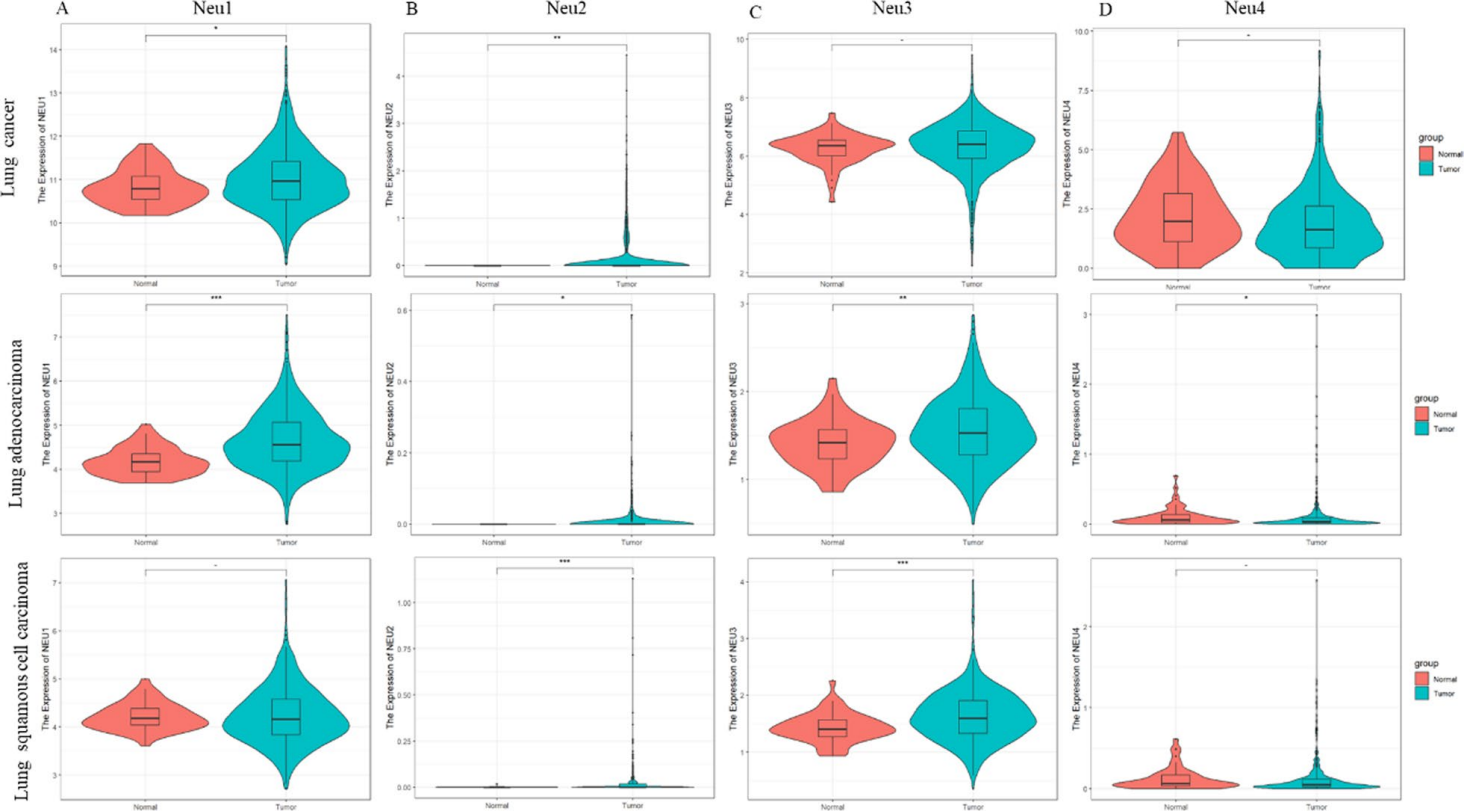
Expression of NEU1 in lung carcinoma in TCGA database. **A** Expression of NEU1 in lung cancer (LC), lung adenocarcinoma (LA) or lung squamous cell carcinoma(LSCC); **B** expression of NEU2 in LC/LA/LSCC; **C** expression of NEU3 in LC/LA/LSCC; **D** expression of NEU4 in LC/LA/LSCC; (lung cancer (n = 1093) lung adenocarcinoma (n = 556), lung squamous cell carcinoma (n = 538) ***p < 0.001, **p < 0.01, *p < 0.05, − p < 1)

NEU-1 exhibited pronounced effects on the development of several cancers by regulated the cancer cells' proliferation and migration, including hepatocellular cancer, pancreatic carcinoma and breast cancer. This sialidase [27, 28]. RNA-seq data of 1093/556/538 patients with lung cancer (LC), lung adenocarcinoma (LA) or lung squamous cell carcinoma (LSCC) were extracted from the TCGA database, and the expression of NEU1 in tumors of lung and paracancer tissue samples was analyzed using R language, as shown in Fig. 2. Higher transcriptional level of NEU1 was found to be remarkably correlated with LC and LA in human based on bioinformatical analysis (P = 0.05 for LC, P = 2.61*10^–16^ for LA), but has no significant change in LSCC (Fig. 2A). NEU2 level all increased in different LC (Fig. 2B). The expression of NEU3 is both remarkably raised in LA or LSCC, except in LC (Fig. 2C), this indicated that NEU3 expression can be used to distinguish LC from other two LA. Although NEU4 expression was only upregulated in LA (Fig. 2D), but its constitutive expression in lung was low, NEU4 was not detected in paracancer tissue of most patient with LA. It suggested that Neu4 was not proper to be an accurate marker of LA. Those results indicated that the different sialidase had specific effects on tumors of lung.

Lung cancer is the most common cancer and the leading cause of cancer-related deaths, including small-cell lung cancer (SCLC) and non-small cell lung cancer (NSCLC) [29, 30]. Several evidence indicates that Neu1 participated in multistage tumorigenesis through binding with matrix metalloproteinase-9 and G protein-coupled receptor tethered to receptor tyrosine kinases (RTKs) and TOLL-like receptor (TLRs) [31, 32]. The p53 tumor suppressor gene mutation highly occurred in lung cancer, involved in promoting cell migration and tumor metastasis [30]. Recent study found that mutant p53 (p53-R273H) promoted NSCLC cell mobility by accelerated NEU1 transcription via activation of AKT signaling [33]. Previous reports demonstrated that Neu1-meidated HA-CD44 binding play an important role in asthma [3], while HA-CD44 binding is also correlated with tumorigenesis or metastasis in lung cancer [34, 35]. It has been proved deglycosylation by neuraminidase induced CD44-HA binding in human lung cancer cell lines [36], but whether NEU1 is the key neuraminidase for HA deglycosylation in lung cancer remains unclear. NEU1 was also identified to be correlated with levels of drug resistance in lung cancer [34, 35]. The association between abnormally high expression of Neu1 and poor prognosis of lung cancer needs further investigation.

## NEU1 and COVID-19

Neu1 is correlated with immune cell activation, including T cells, B cells, and monocytes [36–41]. Upregulation of Neu1 activity in activated murine lymphocytes only increased production of IL-4 and have no effect on other Th1 cytokine such as IFN-γ, while raised the production of IFN-γ in activated human T lymphocytes [42]. Neu1 regulate B cell activation via altered the sialic-acid-binding immunoglobulin-like lectins (Siglecs) CD22 organization in plasma membrane [43, 44]. Endogenous Neu1 sialidase activity is significantly increased during the process of monocytes differentiate into macrophages, which trigger MHC II-enriched compartments [45, 46].

Abnormally high expression of NEU1 interacted with MMP-9, contributed to neutrophil overactivation from COVID-19 patients with severe infections [47]. NEU1 inhibitors (oseltamivir and zanamivir) dampened neutrophil dysfunction and improved infection control as well as host survival in pulmonary infection. SARS-CoV-2 infected human cells through angiotensin-converting enzyme 2 (ACE2) on host plasma. ACE2, as a sialylated glycoprotein, its sialic acids are vital for SARS-CoV-2 infection [48, 49]. Reduced NEU1 activity might aggravate SARS-CoV-2 infectious through promoting excessive lysosomal exocytosis in host cells [50], but this hypothesis needs more clinical and basic evidences to support. In addition, some patients recovered from COVID-19 didn’t produce detectable neutralizing antibodies [51], whether this phenotype is associated the immune dysfunction caused by NEU1 deficiency in cellular response, we should learn it more accurately and deeply. Recent studied revealed that host NEU1 interplay with MMP-9 caused neutrophil overactivation by shedding Sia during severe infections including in sepsis and COVID-19 [47]. The newest report shows that highly glycosylated N protein of SARS-CoV-2 and HCoV-OC43 was regulated by host NEU1. Neu5Ac2en-OAcOMe, a newly selectively Neu1 inhibitor targeted intracellular sialidase, remark reduced HCoV-OC43 and SARS-CoV-2 replication in vitro and rescued mice from HCoV-OC43 infection-induced death [52]. However, it is important to note that the role of NEU1 in COVID-19 is still not fully understood and further research is needed to elucidate its precise mechanisms and implications in the disease.

## NEU1 inhibitor

Sialidases are involved in several human disorders such as metabolic diseases, infectious, lung diseases, kidney diseases, cardiovascular diseases, and cancers. Neuraminidase inhibitors (NAIs) designed for bacterial or viral have different activity against human neuraminidase in treatment of patients with influenza and chronic respiratory disease ([53–58], Table 1). Consistent with this, sialidase inhibitors DANA and oseltamivir (Tamiflu) strongly attenuated pulmonary fibrosis induced by bleomycin in the mouse model [8, 59] C9-pentylamide analogues of DANA show the inhibition potency toward NEU1 [57], C9-butyl-amide-2-deoxy-2,3-dehydro-N-acetylneuraminic acid (C9-BA-DANA), turned out to be the most selective inhibitor for NEU1, inhibited endogenous and ectopically expressed sialidase activity [58, 60]. C5, C9-modified DANA analogues increased potency of DANA analogues against NEU2 [61, 62].C9-triazolyl DANA derivatives exhibited remarkably increased activity for NEU3 [63].C9-4-hydroxymethyltriazolyl DANA (C9-4HMT-DANA) is active against NEU4 [64]. Although these inhibitors might provide potential tools for investigation of the specific role of human NEU isoenzymes in biological systems, but the activity of these compounds in vivo needed further study.

**Table 1:** Summary of Neuraminidase inhibitors (NAIs)

Compound	IC50 of human neuraminidase				References
	Neu1	Neu2	Neu3	Neu4	
Zanamivir	> 500 μM	(7.8 ± 2.0) μM	(4.0 ± 0.6) μM	(47 ± 6) μM	[48, 49]
Oseltamivir	> 500 μM	> 500 μM	> 500 μM	> 500 μM	
Peramivir	> 500 μM	(70 ± 7) mM	> 500 μM	> 500 μM	
2-deoxy-2,3-didehydro-N-acetylneuraminic acid (DANA)	(49 ± 8) μM	(37 ± 6) μM	(7.7 ± 0.8) μM	(8.3 ± 1.0) μM	[50]
Neu5AcN32en	(24 ± 2) μM	(22 ± 3) μM	(4.4 ± 0.9) μM	(5.8 ± 0.8) μM	
C9-butyl-amide-2-deoxy-2,3-dehydro-N-acetylneuraminic acid (C9-BA-DANA)	(3.4 ± 0.2) μM	> 500 μM	(110 ± 40) μM	(220 ± 50) μM	
C9-biphenyltriazolyl DANA(C9-4BPT-DANA)	> 500 μM	(32 ± 5) μM	(0.7 ± 0.1) μM	(0.52 ± 0.1) μM	[51]
C9-4-hydroxymethyltriazolyl DANA(C9-4HMT-DANA)	(620 ± 10) μM	(240 ± 20) μM	(19.7 ± 2.3) μM	(60 ± 20) μM	[52]

Traditional Chinese medicine (TCM) has its unique advantages in the treatment of human diseases. It is an important method to screen the effective drugs for the target of human diseases from TCM. Plenty of Chinese herbs and their extracts also exhibited strong sialidases inhibitory activity, including Lonicerae Japonicae Flos, Scutellariae Radix, *Olyra latifolia* L. leaves, Huanglian Jiedu Decoction and others [65–68]. Our study found that dipsacoside B ameliorated APAP-induced hepatotoxicity by prohibiting Neu1 [69]. The newest evidence supported that salvianolic acid B show strong ability to inhibit Neu1 activity during renal fibrosis development [70]. Interfering peptides (IntPep) targeting the transmembrane (TM) domains 2 of human membrane NEU1 has been proved to disrupt NEU1 dimerization and efficiently block the sialidase activity at the plasma membrane [71]. In a word, Neu1 inhibitors have shown promise as potential therapeutic agents in various diseases and conditions, but further research is needed to fully understand their mechanisms of action and evaluate their efficacy and safety.

## Concluding remarks and future challenges

Numerous studies have provided evidence for the involvement of NEU1 in the pathogenesis of respiratory diseases. NEU1 plays a crucial role in the regulation of glycosylation and is involved in the pathogenesis of various respiratory diseases. NEU-mediated mucin1 extracellular ectodomain (MUC1-ED) desialylation regulates pulmonary collagen deposition, fibrosis, bacterial adhesion, and viral infection. While the molecular mechanism of MUC1-ED desialylation mediated recruitment of NEU1 under Pa infection is still unclear. At the same time, with the advances of medical and measure methods, could MUC1-ED and/or flagellin levels in BALF be a rapid diagnostic assay to identify patients with Pa lung infections independent of bacterial culture or genotyping techniques?

NEU1 and NEU3 are abundant sialidases in the lung. Abnormal expression of these sialidases may be a potent marker to distinguish different tumors of lung, but the specific role of these sialidases and the correlated mechanism involved in development of tumors of lung, is needed more and more strong evidence to support.

Although accumulating evidence demonstrated that neuraminidase inhibitors designed for viral or bacterial shown the inhibitory activity of human NEU1 at cellular or animal levels, the clinical studies of the pharmacology effect of NAIs are rare. The direct interaction between NAIs and NEU1 should be explored by a more reliable method, such as the surface plasmon resonance (SPR) assay, affinity chromatographic methods and other methods. Comprehensive studies on efficacy, safety and toxicity of NAIs in humans are urgently to proceed. Targeting Neu1 may represent a promising therapeutic approach for the treatment of these diseases.

Overall, human NEU1 mediated MUC1 desialylation, human NEU1 modulated immune cell differentiate and activation, contribute to the development and progression of various inflammatory, fibrotic, and fibro-inflammatory human pathologies. Further research focusing on the details of human NEU isoenzymes in biological systems is needed to fully understand the mechanisms underlying the involvement of Neu1 in these conditions and to explore its potential as a therapeutic target for the treatment of respiratory diseases. It is promising that NAIs will be effective treatment of various human respiratory diseases.

## Data Availability

Not applicable.
